# FINANCIAL SATISFACTION AND PERSONALITY IN OLDER ADULTS

**DOI:** 10.1093/geroni/igad104.3040

**Published:** 2023-12-21

**Authors:** Yeon Ji Ryou, Mengya Wang, Peter Martin

**Affiliations:** Iowa State University, Ames, Iowa, United States; Iowa State University, Ames, Iowa, United States; Iowa State University, Ames, Iowa, United States

## Abstract

Financial satisfaction is an essential component of the quality of life among adults 65 years and older. However, little is known about how psychosocial factors, such as personality, might influence financial satisfaction in this age group. To address this gap in knowledge, this study explored the relationship between financial satisfaction, the Big-5 personality traits, and social support. For this study, 1,322 participants answering questions, including the Big-5 personality traits, their financial satisfaction, and social activities, were selected from the 2020 Core Health and Retirement Study (HRS) data. Hierarchical regression analyses were conducted to identify the contributors to financial satisfaction, with control variables such as age, gender, and income in the first model. The Big-5 personality traits and social factors, including negative and positive social support, were added to subsequent models. The results revealed that extraversion and conscientiousness were significantly and positively associated with financial satisfaction, whereas neuroticism and agreeableness demonstrated the opposite relationship. Negative social support was also negatively associated with financial satisfaction, whereas positive social support showed the opposite effect. Participating in social activities was found to be positively associated with financial satisfaction among older adults. Older age, being white, female, married or partnered, and reporting better health were also significant predictors of higher financial satisfaction. These findings have significant implications for financial planning and counseling among older adults. Interventions targeting specific personality traits such as extraversion and engaging in more social activities may be particularly effective for improving financial satisfaction among US older adults.

